# Engineered extracellular vesicles with high collagen-binding affinity present superior *in situ* retention and therapeutic efficacy in tissue repair

**DOI:** 10.7150/thno.70448

**Published:** 2022-08-08

**Authors:** Dake Hao, Lu Lu, Hengyue Song, Yixin Duan, Jianing Chen, Randy Carney, Jian Jian Li, Ping Zhou, Jan Nolta, Kit S. Lam, J. Kent Leach, Diana L Farmer, Alyssa Panitch, Aijun Wang

**Affiliations:** 1 Department of Surgery, University of California Davis, Sacramento, CA 95817, USA.; 2 Institute for Pediatric Regenerative Medicine, Shriners Hospitals for Children Northern California, Sacramento, CA 95817, USA.; 3 Department of Radiation Oncology, University of California Davis, Sacramento, CA 95817, USA.; 4 Department of Biomedical Engineering, University of California Davis, Davis, CA 95616, USA.; 5 Stem Cell Program, Department of Internal Medicine, University of California Davis Medical Center, Sacramento, CA 95817, USA.; 6 Department of Biochemistry and Molecular Medicine, University of California Davis, Sacramento, CA 95817, USA.; 7 Department of Orthopaedic Surgery, School of Medicine, University of California Davis, Sacramento, CA 95817, USA.

**Keywords:** Extracellular vesicle, collagen-binding, *in situ* retention, therapeutic efficacy, tissue repair

## Abstract

Although stem cell-derived extracellular vesicles (EVs) have remarkable therapeutic potential for various diseases, the therapeutic efficacy of EVs is limited due to their degradation and rapid diffusion after administration, hindering their translational applications. Here, we developed a new generation of collagen-binding EVs, by chemically conjugating a collagen-binding peptide SILY to EVs (SILY-EVs), which were designed to bind to collagen in the extracellular matrix (ECM) and form an EV-ECM complex to improve EVs' *in situ* retention and therapeutic efficacy after transplantation.

**Methods:** SILY was conjugated to the surface of mesenchymal stem/stromal cell (MSC)-derived EVs by using click chemistry to construct SILY-EVs. Nanoparticle tracking analysis (NTA), ExoView analysis, cryogenic electron microscopy (cryo-EM) and western-blot analysis were used to characterize the SILY-EVs. Fluorescence imaging (FLI), MTS assay, ELISA and reverse transcription-quantitative polymerase chain reaction (RT-qPCR) were used to evaluate the collagen binding and biological functions of SILY-EVs *in vitro*. In a mouse hind limb ischemia model, the *in vivo* imaging system (IVIS), laser doppler perfusion imaging (LDPI), micro-CT, FLI and RT-qPCR were used to determine the SILY-EV retention, inflammatory response, blood perfusion, gene expression, and tissue regeneration.

**Results:** *In vitro*, the SILY conjugation significantly enhanced EV adhesion to the collagen surface and did not alter the EVs' biological functions. In the mouse hind limb ischemia model, SILY-EVs presented longer *in situ* retention, suppressed inflammatory responses, and significantly augmented muscle regeneration and vascularization, compared to the unmodified EVs.

**Conclusion:** With the broad distribution of collagen in various tissues and organs, SILY-EVs hold promise to improve the therapeutic efficacy of EV-mediated treatment in a wide range of diseases and disorders. Moreover, SILY-EVs possess the potential to functionalize collagen-based biomaterials and deliver therapeutic agents for regenerative medicine applications.

## Introduction

Extracellular vesicles (EVs) are cell-secreted and lipid-bound vesicles functioning in intercellular communication by transferring biological information 1, 2. EVs carry cellular biological functional cargo, including DNA, RNA, and proteins for an array of physiological capacities, including tissue damage response and regeneration. EVs also hold the advantage over cell-based therapy due to their special properties, including low toxicity and immunogenicity, biodegradability, ability to encapsulate endogenous biologically active molecules, and capacity to cross the blood-brain barrier (BBB) 3-5. Indeed, EVs have shown potential benefits in various diseases based their multiple regenerative capacities 6-12. EVs in the free form can be delivered to the injured sites via diverse routes 13, 14. Notably, local injection of EVs directly into the injured site is one of the most common routes of EV delivery 15, 16. However, due to the complex multiple-phase process during the recovery of the injured tissue, the locally injected free EVs have difficulties in remaining at the injured site, resulting in low therapeutic efficacy 16-19. Recently, EV immobilization within biomaterials has been developed to improve retention of locally delivered EVs at the injured sites and promote tissue repair 20-23. We previously developed an approach to immobilize EVs onto electrospun polymer biomaterials and showed improved vascularization of the biomaterial scaffolds 24. However, biomaterials are not always appropriate for use in treating various diseases, and moreover, selecting, optimizing, and manufacturing biomaterial scaffolds with adequate properties for EV delivery is a lengthy process. Hence, it is imperative to design and engineer a new generation of EVs that can be easily applied in clinical applications and can persist for a longer local retention* in vivo*.

Currently, different targeting molecules have been applied to engineer EVs and improve their delivery efficiency and therapeutic capacity 25-27. However, the current EVs modified with specific cell/tissue-targeting peptide is only designed to treat the specific disease rather than a broad array of applications 28-30. Extracellular matrix (ECM) is the basic substance that surrounds the resident cells in tissues and provides structural and biochemical support for cell and tissue development 31, 32. Therefore, it makes sense to engineer a new generation of EVs that could bind to ECM and demonstrate superior *in situ* retention, which will be applied to improve the therapeutic efficiency for a wide range of diseases.

Collagen is the main structural protein in the ECM present within all tissue and organs 33, 34. Furthermore, collagen accumulates in tissue in response to injury 35-37. Therefore, collagen serves as a potential target to aid in retaining and enriching EVs to enhance their reparative efficacy for injuries. Also, it is known that physiologically native EVs actively interact with the ECM 38 and the EV-ECM complex is mediating significant biological functions of EVs 39. It has recently been identified that matrix bound vesicles (MBVs) are mediating significant regenerative functions 40-42, which highlights the importance of the EV-ECM structural complex. We investigated a collagen-binding peptide SILY, that has shown a high binding affinity to collagen 33, 43. In this study, to mimic the stable EV-ECM complex and the physiological interactions between the EVs and ECM, we developed a mild, rapid and safe approach to conjugate SILY to the surface of mesenchymal stem cell (MSC)-derived EVs by using click chemistry to construct a new generation of collagen-binding EVs (SILY-EVs) that possess superior capability to bind to collagen backbone of the ECM to form the EV-ECM complex. In a mouse hind limb ischemia model, the SILY-EVs presented longer *in situ* retention, inhibited inflammatory responses, and significantly augmented muscle tissue repair and revascularization, compared to the unmodified EVs.

## Results

### Preparation and characterization of SILY-EVs

**Figure 1:**
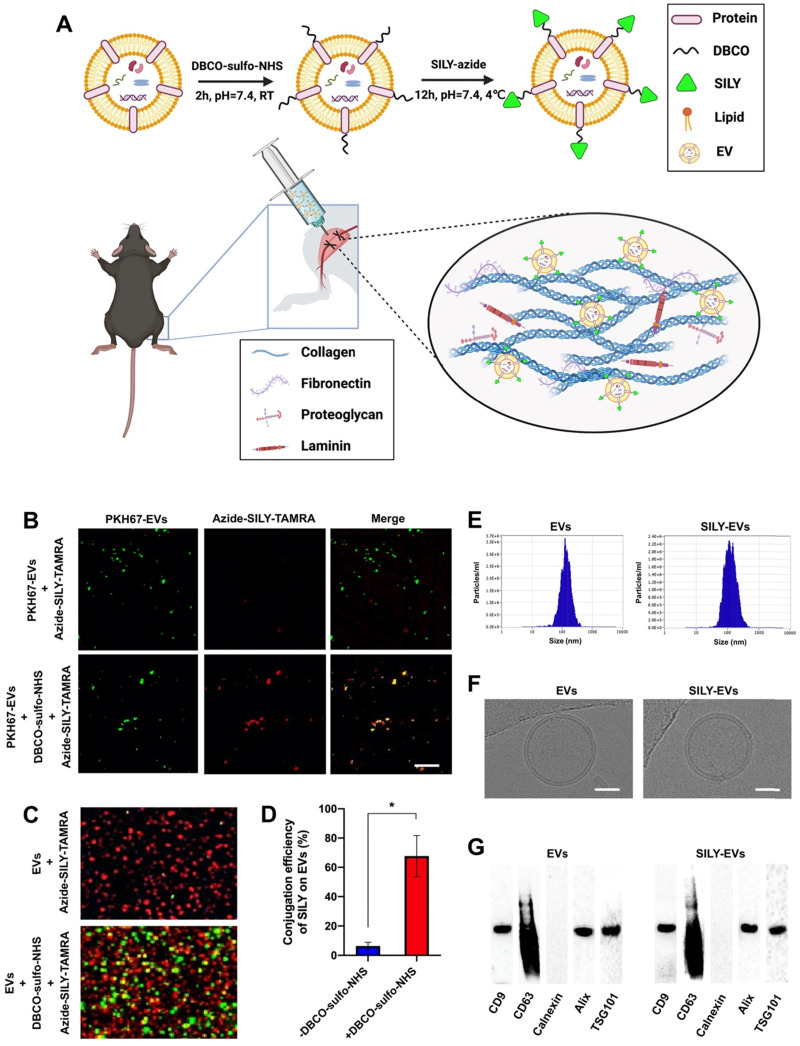
Preparation and characterization of SILY-EVs. (A) Schematic diagram of the study design. (B) Evaluation of the SILY-EV conjugation process. Scale bar = 2 μm. (C) ExoView images of SILY-EV conjugation. Red indicated EVs stained with CD63. Green indicated SILY labeled with TAMRA. (D) Quantification of conjugation efficiency of SILY on EVs via ExoView. (E) Size distributions of EVs and SILY-EVs based on NTA measurements. (F) Cryo-EM images of EVs and SILY-EVs. Scale bar = 50 nm. (G) Western-blot analysis of EVs and SILY-EVs.

MSC-derived EVs have shown promising regenerative potential, including promoting angiogenesis and suppressing inflammation and cell apoptosis 44, 45. Previously, we demonstrated that the human placental MSC-derived EVs (PMSC-EVs) possessed strong pro-angiogenic capacity and inhibited endothelial cell (EC) apoptosis 24. Here, we constructed SILY-EVs by conjugating the SILY peptide to the PMSC-EV surface via a two-step reaction (**Figure 1A**). First, DBCO groups were introduced to the EV surface by linking EVs with DBCO-sulfo-NHS. The NHS groups were conjugated to the amine groups present on the surface proteins of EVs to form covalent bonds. Secondly, the DBCO groups on the EVs were then combined with azide-SILY to form stable triazole linkages using copper-free click chemistry. To assess the SILY conjugation qualitatively, PKH67-labeled EVs were conjugated to the TAMRA (a red dye) labeled azide-SILY with or without DBCO-sulfo-NHS. The results showed that the TAMRA and the PKH67 merged efficiently in the group with DBCO-sulfo-NHS. In contrast, almost no TAMRA and PKH67 merged in the group without DBCO-sulfo-NHS (**Figure 1B**). The results indicated the SILY was successfully conjugated to the EV surface by using the conjugation approach established in this study. ExoView tetraspanin kit assay was used to further quantificationally measure the SILY conjugation efficiency on EVs. The results showed the SILY conjugation efficiency on EVs was about 70% (**Figure 1C-D**).

The EVs and SILY-EVs were characterized by using nanoparticle tracking analysis (NTA), cryogenic electron microscopy (cryo-EM), BCA assay and western-blot analysis. The NTA results showed that the EVs had diameters of 119.6-133.6 nm and the SILY-EVs had diameters of 114.2-147.6 nm (**Figure 1E**), which indicated the SILY conjugation approach did not affect the size distribution of the EVs. The cryo-EM results exhibited intact structure and similar round morphology of both the EVs and SILY-EVs (**Figure 1F**), confirming the SILY conjugation approach maintained the integrity of EV structure. The results of the BCA assay showed no significant difference in the protein content between the same number of EVs and SILY-EVs, indicating the SILY conjugation did not alter the protein content of EVs (**Figure S1**). The western-blot results showed both EVs and SILY-EVs expressed the typical EV markers, such as CD9, CD63, Alix and TSG101, and did not express endoplasmic reticulum marker calnexin (**Figure 1G**), which indicated the SILY conjugation approach designed in this study did not alter the expression of typical EV markers.

To evaluate cellular uptake of EVs and SILY-EVs, DiD-labeled EVs or SILY-EVs were incubated with human endothelial colony forming cells (HECFCs) that is crucial for vascularization 46-48. The results showed the percentage of cells with internalized EVs or SILY-EVs was about 30% after incubation for 4 h, and the percentage increased to over 90% after incubation for 16 h. There was no significant difference between the EVs and SILY-EVs. These results indicated that the percentage of cells with internalized EVs or SILY-EVs increased with time, and the SILY conjugation approach maintained the cellular uptake efficiency of EVs (**Figure S2**). To avoid the effects of the DiD dye background, we further evaluated the cellular uptake of EVs or SILY-EVs using a mixture of DiD-labelled (10%) and non-labelled (90%) EVs or SILY-EVs, and the signal of EVs or SILY-EVs untaken by the HECFCs was significantly decreased, compared to the all DiD-labelled (100%) EVs or SILY-EVs (**Figure S2**), which was consistent with the previous results.

We further performed the *in vitro* time-course experiments to evaluate the kinetics of EVs and SILY-EVs internalized in HECFCs. After another 48-h culture in the medium without EVs and SILY-EVs, both the EVs and SILY-EVs internalized in HECFCs decreased, and there was no significant difference between EVs and SILY-EVs (**Figure S3**). These results demonstrated the SILY conjugation approach did not alter the degradation rate of EVs internalized in cells.

Tube formation assay, cell proliferation assay and Reverse transcription-quantitative polymerase chain reaction (RT-qPCR) were performed to further evaluate the effects of the SILY conjugation approach on EVs' biological functions. The results showed no significant difference on HECFC tube formation, HECFC proliferation and expression of pro-angiogenic and inflammatory genes (**Figure S4**), confirming the SILY conjugation approach did not affect the EVs' biological functions.

### SILY-EVs exhibited higher binding affinity to collagen, subsequently suppressed inflammation and cell apoptosis, and improved pro-angiogenic capacity

**Figure 2:**
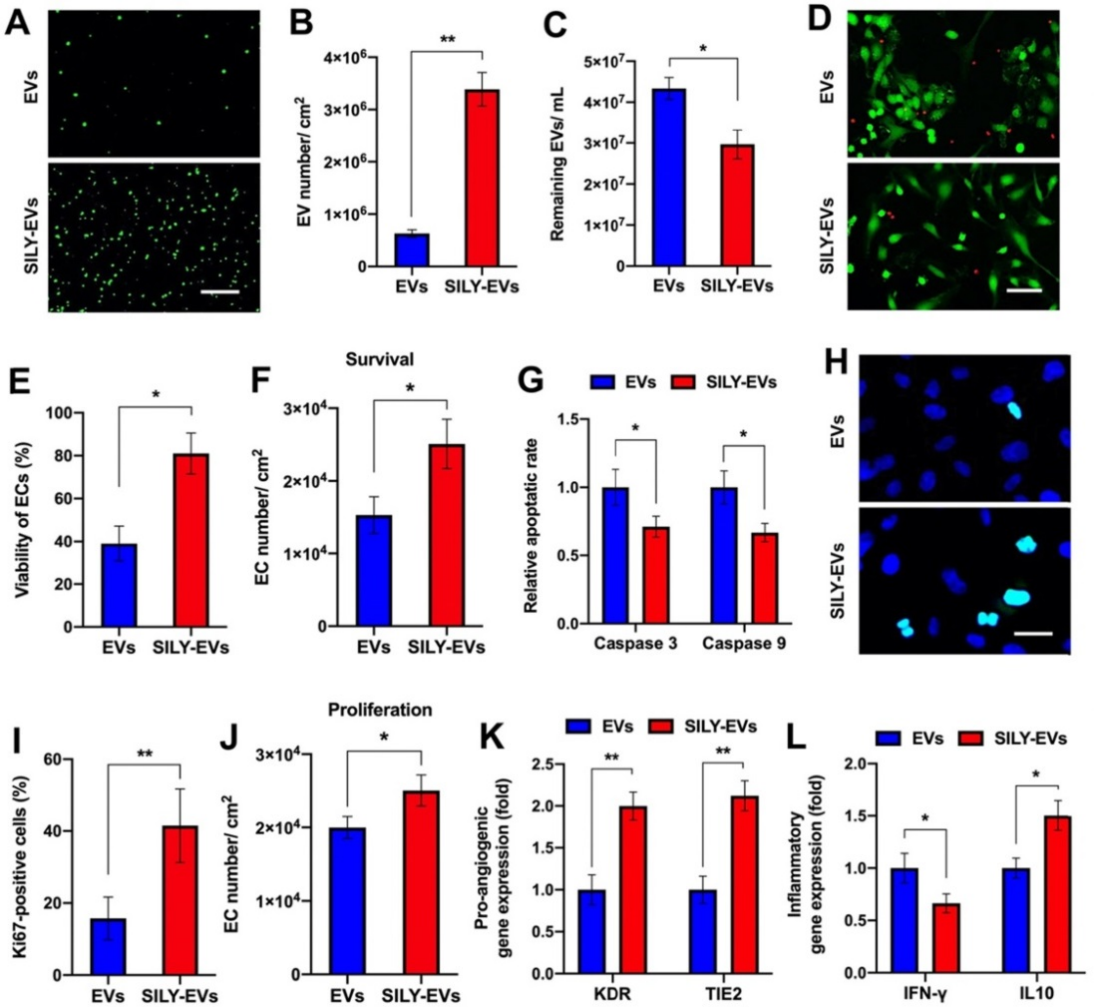
Binding ability of SILY-EVs to collagen and their effects on biological functions. (A) Images of attached PKH67-labeled EVs or SILY-EVs on collagen surface. Scale bar = 3 μm. (B) Quantification of the numbers of EVs or SILY-EVs attached to the collagen surface. (C) Quantification of the numbers of the EVs or SILY-EVs unattached to the collagen surface by using NTA. (D) Images and (E) quantification of HECFC viability, green for live cells and red for dead cells, cultured on the collagen surfaces decorated with EVs or SILY-EVs under the ischemic simulated environment. Scale bar = 50 μm. (F) Survival of HECFCs and (G) expression of caspase 3 and caspase 9 in HECFCs cultured on the collagen surfaces decorated with EVs or SILY-EVs under the ischemic simulated environment. (H) Images and (I) quantification of Ki67 positive HECFCs cultured on the collagen surfaces decorated with EVs or SILY-EVs. Scale bar = 10 μm. (J) Proliferation of HECFCs cultured on the collagen surfaces decorated with EVs or SILY-EVs. (K) Expression of pro-angiogenic genes (KDR and TIE2) in HECFCs cultured on the collagen surfaces decorated with EVs or SILY-EVs. (L) Expression of IFN-γ and IL10 related to inflammatory responses in the LPS-stimulated human macrophage-like THP-1 cells cultured on the collagen surfaces decorated with EVs or SILY-EVs. Data are expressed as mean ± standard deviation: *p < 0.05, **p < 0.01 (n = 6).

To evaluate the binding ability of SILY-EVs to collagen, we coated the culture surface with type I collagen and investigated the attachment of EVs or SILY-EVs on the collagen surface via imaging and NTA. Both the results of imaging and NTA showed that the SILY conjugation significantly improved EV attachment on the type I collagen surface (**Figure 2A-C**). Besides type I collagen, type III collagen is the second most abundant collagen in the body 49. We further evaluated the binding ability of SILY-EVs on type III collagen. The results showed more SILY-EVs bound on the type III collagen compared to the EVs, but there was no significant difference between two groups (**Figure S5**). Based on the attachment of EVs or SILY-EVs on the type I collagen surface, we further evaluated the effects of the collagen surfaces decorated with EVs or SILY-EVs on biological functions. In the cell survival study, the ischemic simulated environment was set up as basic endothelial basal medium-2 (EBM-2) with 1% fetal bovine serum (FBS) and low concentration growth factors at 1% O_2_, 37^◦^C and 5% CO_2_. In the *in vitro* ischemic simulated environment, the collagen surface decorated with SILY-EVs significantly improved HECFC viability (**Figure 2D-E**) and survival (**Figure 2F**) and suppressed HECFC apoptosis by decreasing the expression of Caspase 3 and Caspase 9 (**Figure 2G**), compared to the collagen surface decorated with EVs. Additionally, the collagen surface decorated with SILY-EVs significantly improved Ki67 expression in HECFCs (**Figure 2H-I**) and HECFC proliferation (**Figure 2J**), compared to the collagen surface decorated with EVs. Moreover, the expression of angiogenic genes, including KDR and TIE2, was significantly increased in the HECFCs cultured on the collagen surface decorated with SILY-EVs, compared to the HECFCs cultured on the collagen surface decorated with EVs (**Figure 2K**). In the inflammatory response study, compared to the collagen surface decorated with EVs, the collagen surface decorated with SILY-EVs significantly decreased the expression of Th1-related gene IFN-γ and increased the expression of Th2-related gene IL10 (**Figure 2L**) in lipopolysaccharide (LPS)-stimulated human macrophage-like THP-1 cells. These results demonstrated that the SILY conjugation increased the number of EVs attached to the collagen surface, resulting in enhanced angiogenic capacities and suppressed inflammatory responses. Hence, this SILY conjugation approach holds promise to promote EV regenerative functions *in vivo* by improving EV retention.

### SILY-EVs possessed prolonged retention in a mouse hind limb ischemia model

**Figure 3:**
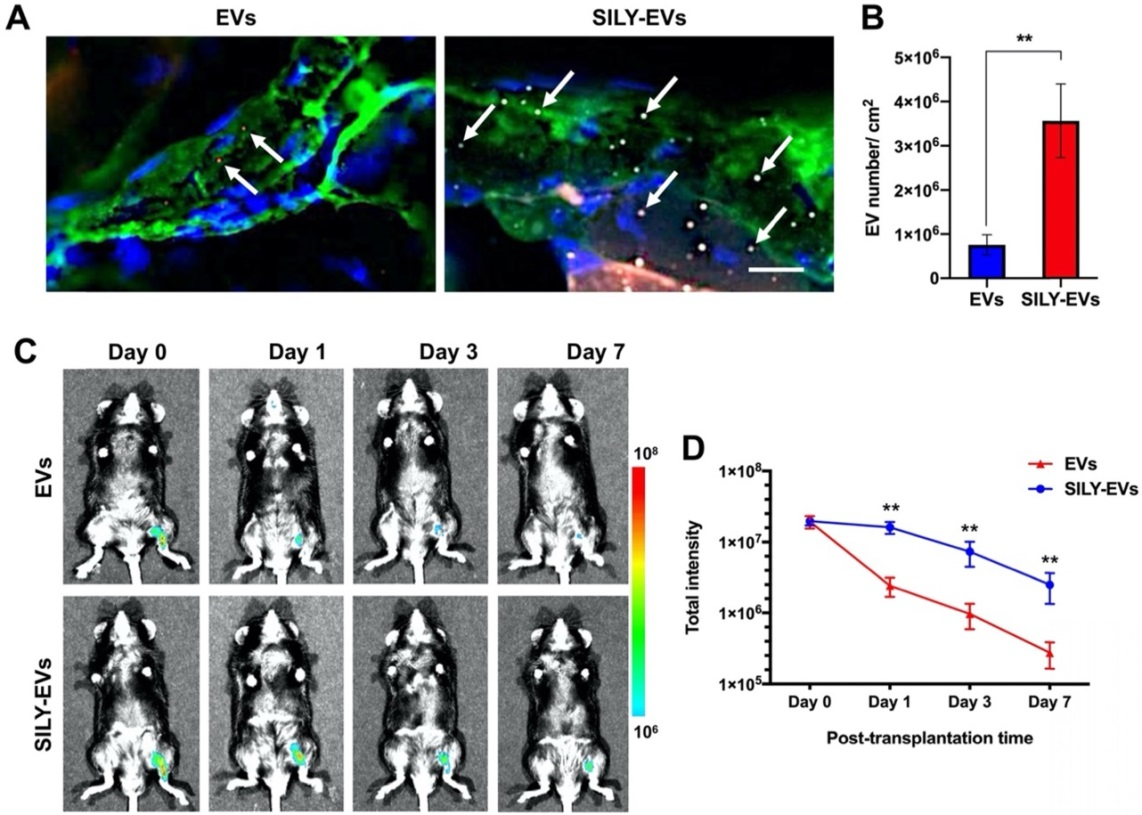
Retention of SILY-EVs in a mouse hind limb ischemia model. (A) *Ex vivo* evaluation for retention of EVs and SILY-EVs on the ischemic hind limb tissue section via binding to collagen. Green indicated collagen, blue indicated nucleus, red indicated DiD-labeled EVs, and gray indicated TAMRA-labeled SILY. The white arrows indicated EVs or SILY-EVs attached on the collagen of the ischemic hind limb tissue section. Scale bar = 10 μm. (B) Quantification of the EVs and SILY-EVs attached on the collagen of the ischemic hind limb tissue section. (C) Representative IVIS images of the retention of EVs or SILY-EVs at different time points after transplantation in the mouse hind limb ischemia model. (D) Quantification of the fluorescence intensity in EV group and SILY-EV group after transplantation in the mouse hind limb ischemia model. Data are expressed as mean ± standard deviation: **p < 0.01 (n = 6).

Before EVs were transplanted into the mouse hind limb ischemia model, we performed the *ex vivo* study to evaluate whether the retention of SILY-EVs was significantly higher on the ischemic hind limb tissue via binding to collagen, compared to the unmodified EVs. The results showed abundant type I collagen expressed in the ischemic hind limb tissue, and the retention of SILY-EVs was significantly improved on the ischemic hind limb tissue via binding to collagen compared to the EVs (**Figure 3A-B**). We further evaluated the retention of EVs or SILY-EVs on the ischemic hind limb tissue sections after incubating for 1, 3 or 7 days. The results showed the retention of SILY-EVs was significantly higher on the ischemic hind limb tissue via binding to collagen at day 1, 3 and 7, compared to the EVs, and the numbers of both EVs and SILY-EVs were lower with time (**Figure S6**).

Subsequently, the EVs or SILY-EVs were transplanted into the mouse ischemic hind limb via intramuscular injection to evaluate the retention of EVs or SILY-EVs at different time points. To characterize the EV biodistribution after intramuscular administration, whole animal imaging was conducted using the *In Vivo* Imaging System (IVIS) to visualize the presence of the EVs in the entire body of the animals. IVIS results showed that on day 1 after injection, the signal in the EV group decreased sharply at the injection site, whereas the signal in SILY-EV group persisted. On day 7 after injection, the signal in the EV group was almost undetectable, whereas the signal was still visible in the SILY-EV group (**Figure 3C-D**). These results demonstrated that SILY-EVs possessed longer retention *in vivo,* compared to the unmodified EVs. This longer retention of SILY-EVs may lead to improved therapeutic efficacy in disease treatment. Moreover, we did not find any injected EVs in other internal organs.

### SILY-EVs promoted blood perfusion and vascular remodeling in the mouse hind limb ischemia model

**Figure 4:**
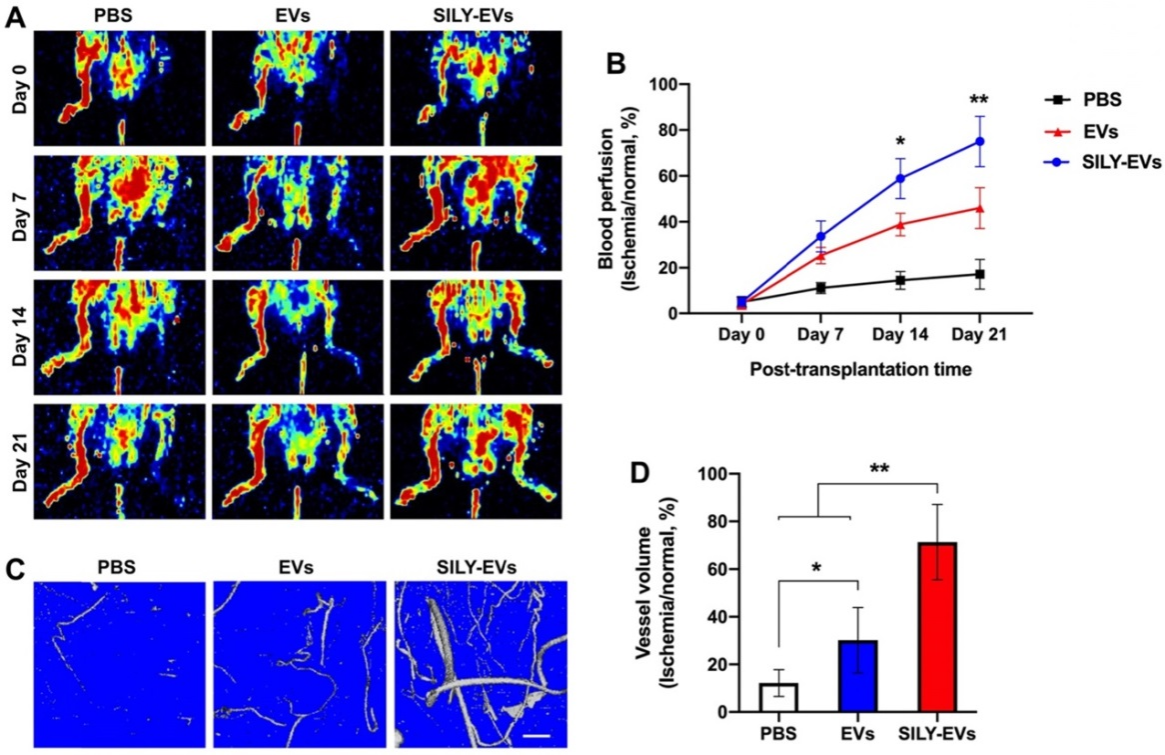
Effect of SILY-EVs on blood perfusion and vascular remodeling in the mouse hind limb ischemia model. (A) Representative LDPI of blood perfusion. (B) Quantification of the blood perfusion. (C) Micro-CT images of blood vessel. Scale bar = 1 mm. (D) Quantification of the blood vessel volume. Data are expressed as mean ± standard deviation: *p < 0.05, **p < 0.01 (n = 6).

To further determine the effects of SILY-EVs on the ischemic disease, EVs or SILY-EVs were intramuscularly injected into the mouse ischemic hind limb on day 1 after surgery. After the injection of EVs and SILY-EVs, all animals were closely monitored for up to 21 days to comprehensively evaluate for safety, especially toxicity and tumorigenicity. Daily visual inspection of the animal's condition includes mortality, clinical presentation, body weight, and consumption of food. No obvious toxicity and tumorigenicity was observed in any animals during the entire study period. The blood perfusion was evaluated by using Laser Doppler perfusion imaging (LDPI). Although both EV and SILY-EV treatments increased blood perfusion compared to the PBS treatment on days 7, 14, and 21 after delivery, blood perfusion in the SILY-EV treatment group was significantly improved compared to the EV treatment group on days 14 and 21 after surgery (**Figure 4A-B**). To assess the effect of SILY-EVs on vascular remodeling in the mouse ischemic hind limbs, mice treated with PBS, EVs or SILY-EVs were injected with a contrast media, Microfil MV-120, into the left ventricle on day 21 after surgery. Large and branching vessels in the ischemic limbs were detected using micro-CT imaging (**Figure 4C**). The total vessel volume in the mouse ischemic limbs in both the EV treatment group and SILY-EV treatment group were significantly improved, compared to that in the PBS treatment group. Furthermore, the SILY-EV treatment significantly enhanced the total vessel volume compared to the EV treatment (**Figure 4D**). These results demonstrated that SILY-EVs promised greater capability to promote vascular remodeling and blood reperfusion in the ischemic disease model compared to conventional unmodified EVs.

### SILY-EVs inhibited inflammatory response and promoted M2 polarization of macrophages in the mouse hind limb ischemia model

**Figure 5:**
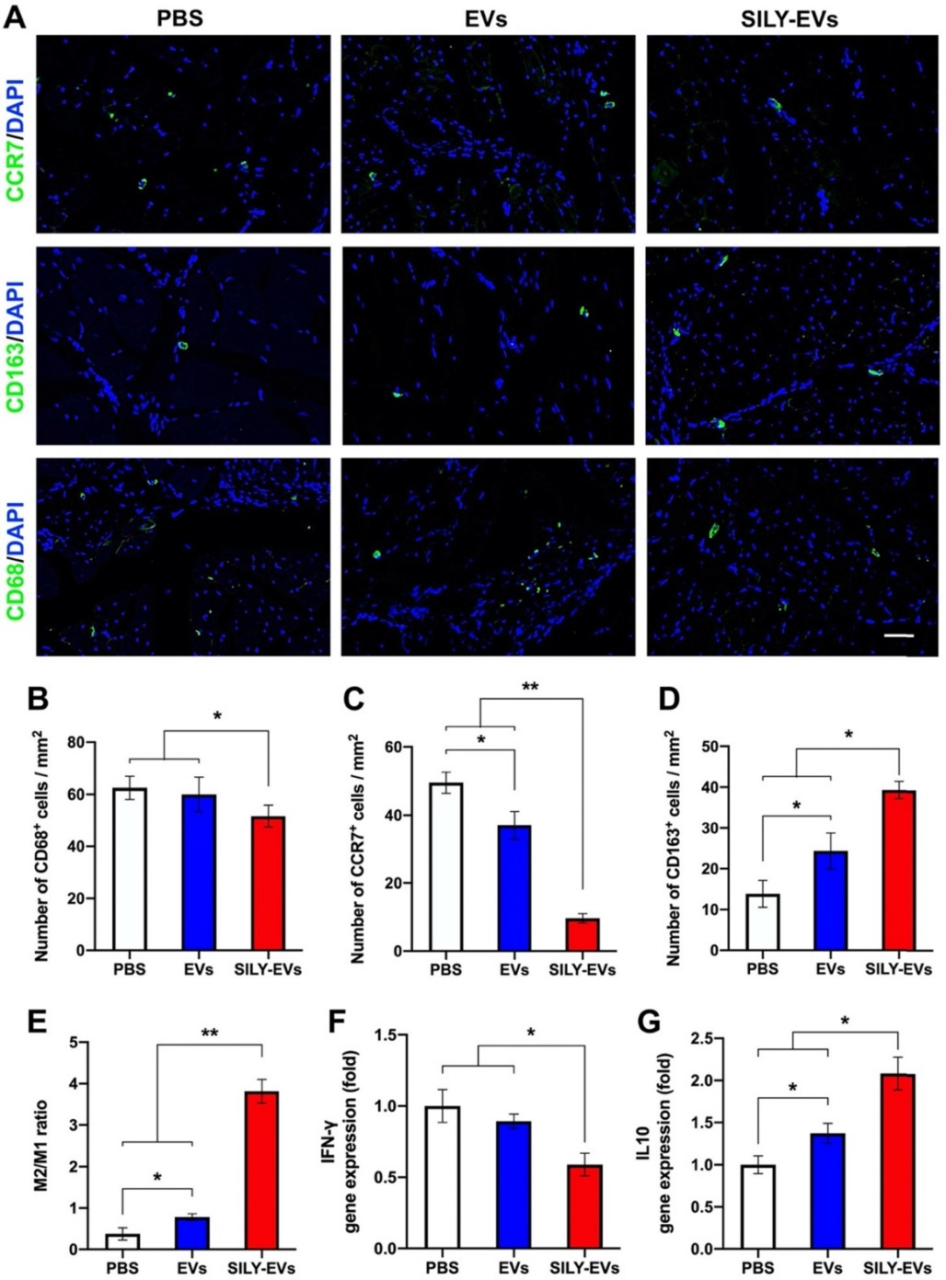
Effects of SILY-EVs on inflammatory response and macrophage polarization in the mouse ischemic hind limb. (A) Representative immunofluorescence staining of CCR7^+^ M1 macrophages, CD163^+^ M2 macrophages and CD68^+^ macrophages. Scale bar = 50 μm. Quantification of the number of (B) CD68^+^ macrophages, (C) CCR7^+^ M1 macrophages, (D) CD163^+^ M2 macrophages and (E) M2/M1 ratio in the ischemic hind limb. Gene expression of (F) IFN-γ and (G) IL10 in different treatment groups. Data are expressed as mean ± standard deviation: *p < 0.05, **p < 0.01 (n = 6).

The role of macrophages in tissue healing and repair is correlated with their ability to polarize between pro-inflammatory and anti-inflammatory phenotypes 50-52. M1 macrophages are capable of high antigen presentation and promote Th1 differentiation of lymphocytes that produce pro-inflammatory cytokines in response to intracellular pathogens. M2 macrophages release anti-inflammatory cytokines and display a high level of iron export, aiding in tissue remodeling 53-55. Hence, understanding the control of the M2/M1 ratio through the modulation of microenvironmental cues will be a key step to enhance positive tissue remodeling, integration, and regeneration. Here, we evaluated the effects of SILY-EVs on the inflammatory response and the control of M2/M1 ratio in the mouse ischemic hind limb. The slides of ischemic hind limb tissue were investigated for the macrophage marker CD68, M1 macrophage marker CCR7, and M2 macrophage marker CD163 at day 7 after EV or SILY-EV injection. CD68^+^ macrophages were significantly reduced in the SILY-EVs treatment group compared to the EV and PBS treatment groups (**Figure 5A-B**). CCR7^+^ M1 macrophages were dramatically increased in the PBS and EV treatment groups and were attenuated in the SILY-EV treatment groups (**Figure 5A, C**). CD163^+^ M2 macrophages were significantly higher in the SILY-EV treatment group, compared to the other two groups (**Figure 5A, D**). Although both the EV treatment group and SILY-EV treatment group improved the M2/M1-like macrophage ratio, the SILY-EV treatment significantly and substantially promoted the macrophage polarization from the M1 to the M2, compared to the EV treatment and PBS treatment (**Figure 5E**). Additionally, we further determined the gene expression related to inflammatory response, we found SILY-EV treatment significantly decreased the expression of Th1-related gene IFN-γ (**Figure 5F**) and increased the expression of Th2-related gene IL10 (**Figure 5G**), compared to the EV and PBS treatments. All of these results demonstrated that compared to the EV treatment, SILY-EV treatment had a greater impact on suppressing the inflammatory response at the injure area and promoting M2 polarization of macrophages, which are vital in promoting tissue regeneration and healing.

### SILY-EVs accelerated muscle tissue repair and revascularization in the mouse hind limb ischemia model

**Figure 6:**
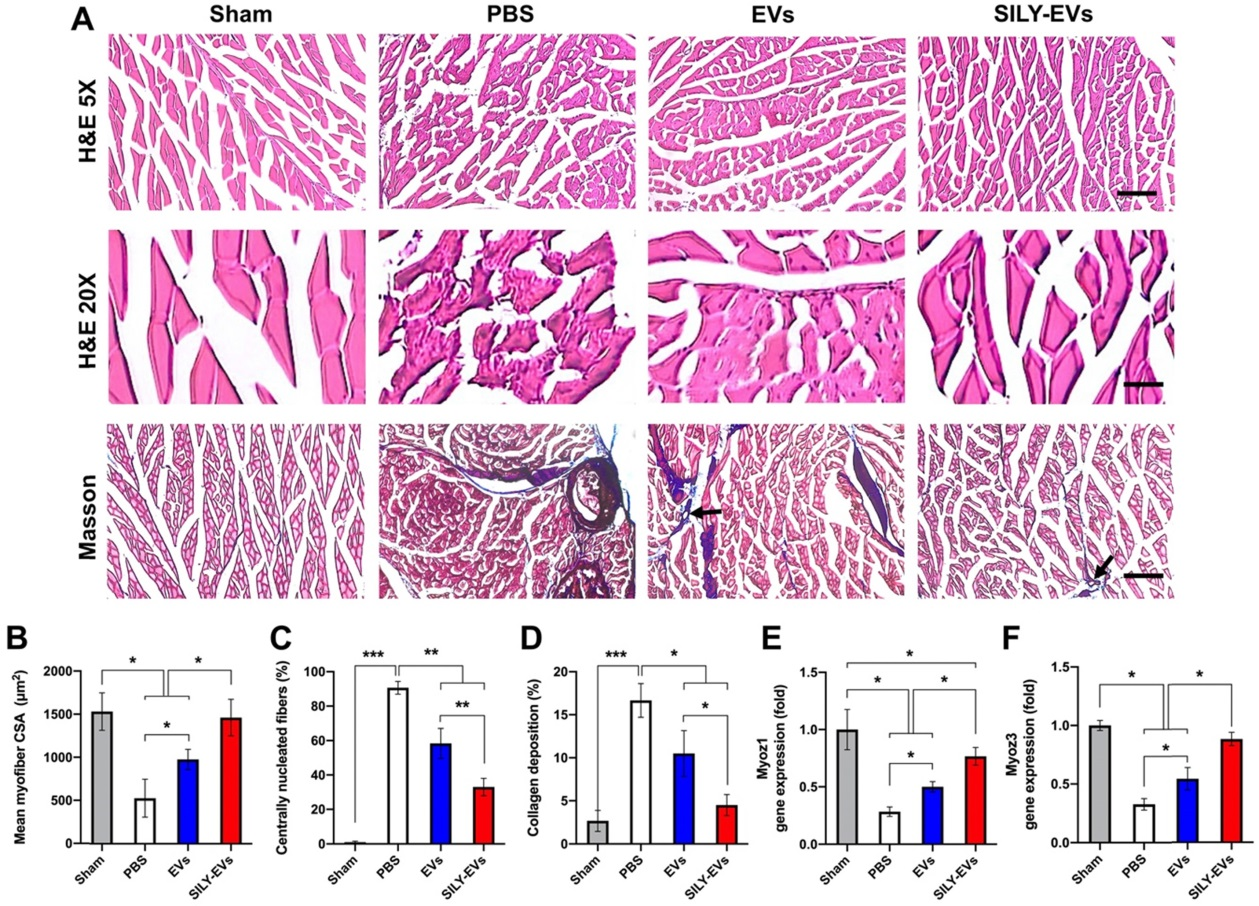
Effects of SILY-EVs on muscle repair in the ischemic hind limb. (A) Representative hematoxylin and eosin (H&E) and masson trichrome staining images of ischemic muscles treated with PBS, EVs, and SILY-EVs. The arrows indicated the vascular structures. Scale bar = 100 μm in H&E 5X images, 20 μm in H&E 20X images, and 100 μm in Masson images. Quantification of (B) average myofiber size, (C) percentage of centrally nucleated myofibers, and (D) area of collagen deposition. Gene expression of (E) Myoz1 and (F) Myoz3 in different treatment groups. Data are expressed as mean ± standard deviation: *p < 0.05, **p < 0.01, ***p < 0.001 (n = 6).

Having established improved retention, and enhanced polarization of macrophages to an M2 phenotype, we next evaluated the effects of SILY-EVs on ischemic muscle tissue repair. Histologic analysis confirmed significant myofiber augmentation in size (**Figure 6A-B**), reduction of centrally located nuclei (**Figure 6A, C**) and collagen deposition (**Figure 6A, D**) in the SILY-EV treatment group, compared to the EV and PBS treatment groups, suggesting that SILY-EVs possess a greater capacity for muscle regeneration compared to EVs. Moreover, we further determined the gene expression related to muscle regeneration, we found SILY-EV treatment significantly increased the expression of Myoz1 (**Figure 6E**) and Myoz3 (**Figure 6F**), compared to the EV and PBS treatments.

**Figure 7:**
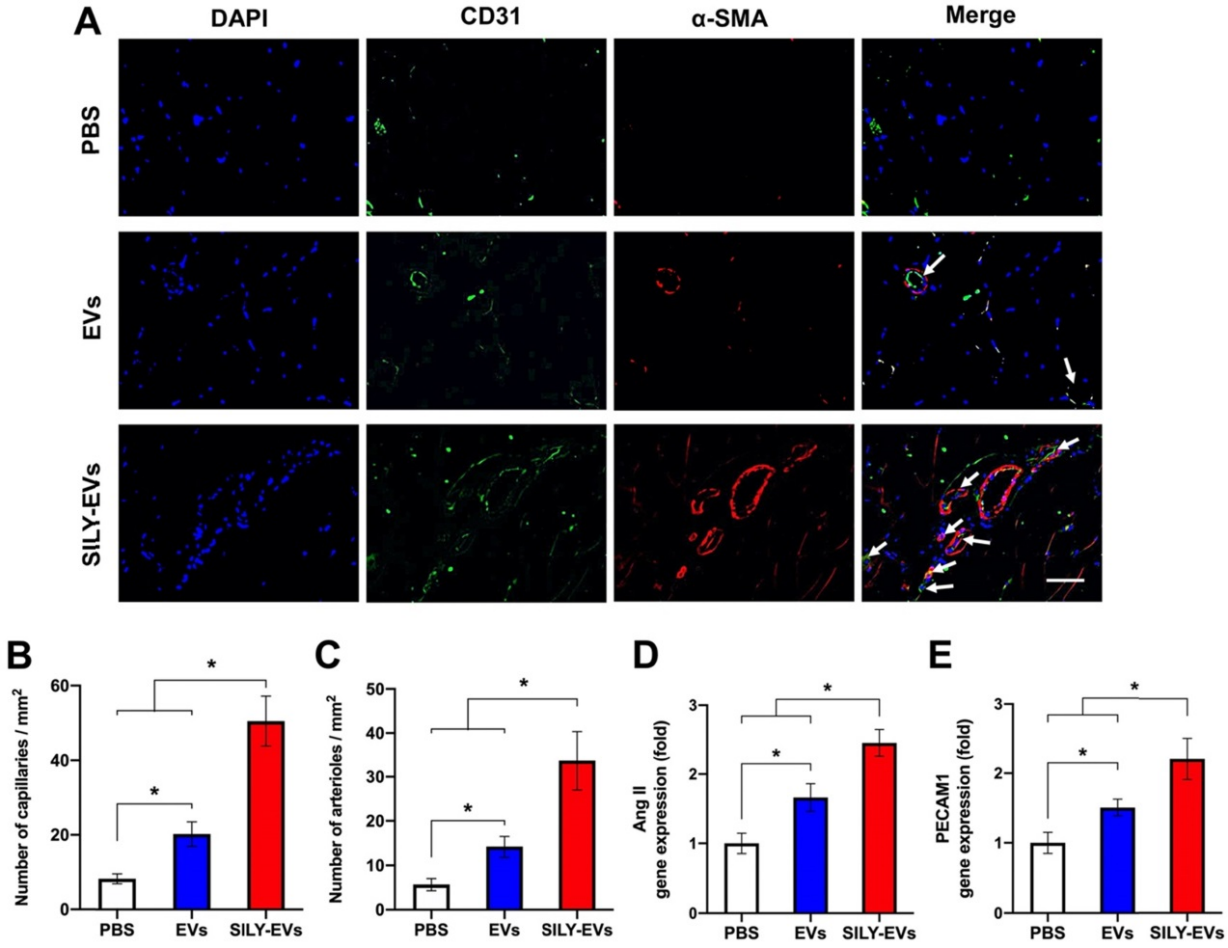
Effects of SILY-EVs on revascularization in the ischemic hind limb. (A) Representative immunofluorescence staining of CD31 and α-SMA. The arrows indicated the capillaries. Scale bar = 15 μm. Quantification of the density of (B) capillaries and (C) arterioles. Expression of angiogenic genes, (D) ANG II and (E) PECAM1, in different treatment groups. Data are expressed as mean ± standard deviation: *p < 0.05 (n = 6).

To evaluate the effects of SILY-EVs on revascularization, we performed CD31 staining for capillaries and α-smooth muscle actin (α-SMA) staining for the arterioles. In agreement with LDPI measurements, the results confirmed that the SILY-EV treatment significantly improved the density of capillaries (**Figure 7A-B**) and arterioles (**Figure 7A, C**), compared to the EV and PBS treatments. In addition, we further determined the expression of angiogenic genes, ANG II and PECAM1, in different treatment groups, and the results showed the SILY-EV treatment significantly increased the expression of ANG II (**Figure 7D**) and PECAM1 (**Figure 7E**), compared to the EV and PBS treatments. These data indicate that the SILY-EVs possess a better regenerative potential on revascularization compared to the EVs. Overall, although EVs were positive regulators in post ischemic recovery in the mouse hind limb ischemia model, the SILY-EVs showed stronger healing efficacy.

## Discussion

Although the emergence of EVs has revealed promising potentials in various tissue repair, the applications of free EVs still face many challenges, especially their rapid clearance *in vivo* 56. Notably, the local injection route is widely used to deliver the EVs to the injured sites for tissue repair; however, the locally injected free EVs always present low retention and poor therapeutic efficacy 15. To address this problem, in this study, we developed a new generation of collagen-binding EVs, SILY-EVs, by conjugating the collagen-binding peptide SILY on the EV surface. After local injection, the SILY-EVs presented superior retention *in vivo* (**Figure 3C-D**) via bound to the collagen in ECM to form the stable EV-ECM complex, resulting in significantly improved therapeutic efficacy in the hind limb ischemia model, compared to the locally injected free EVs (**Figure 4-6**).

Compared to many previous EV surface engineering approaches 5, 57, 58, in this study, a mild, rapid and safe EV surface engineering strategy was established by using the biocompatible copper-free click chemistry that has been applied in various animal models without apparent toxicity and physiological perturbation 59-61. SILY-EVs showed no apparent toxicity nor tumorigenicity during the entire animal study period. In addition, the whole conjugation process is completed in the neutral solution within hours and did not alter the size, structure, integrity, contents, and biological activities of EVs (**Figure 1, Figure S1, Figure S4**). Moreover, this engineering strategy is suitable for conjugating multiple molecules with disparate characteristics to EVs derived from different media, and the densities of molecules can be controlled by adjusting the ratio of the reactants. In future studies, as we further develop SILY-EVs towards clinical translation, we will further perform a detailed toxicological evaluation at the endpoint, which will include full clinical evaluation such as hematology, blood chemistry, organ weights, and full pathology, including gross and histopathology on all tissues and organs. We will also collect the blood to evaluate systemic inflammation after the treatment. Furthermore, a sham-operated group should be included in the future studies, which could be a control group to further interpretate the rationale of the hind limb ischemia model established in this study.

In this study, the SILY-EVs locally injected into the injured site could contribute to sustained tissue repair in a variety of ways. Firstly, SILY-EVs bound to collagen in ECM could still interact directly with the surrounding cells via EV-cell fusion. Secondly, SILY-collagen binding is a non-covalent molecular interaction-based binding. Therefore, there will be a dynamic release of EVs from ECM collagen which will then interact with the surrounding cells. Lastly, EV cargos, e.g., miRNAs and proteins, can be released from EVs which can directly interact with the cells in the local environment. Moreover, since collagen is distributed within most tissues 33, 62, the SILY-EVs could be widely used for various tissue repairs.

Collagen-based biomaterials have been widely used in clinical applications, and the EVs have also been developed to functionalize the biomaterial scaffolds in the tissue regeneration field 15, 24, 63, 64. The SILY conjugation approach established in this study could be used to optimize and improve the efficiency of EV modification on collagen-based biomaterials. Moreover, many studies have demonstrated that EVs could overcome the barriers of artificial nanoparticles 3, 65-67 and act as the effective delivery platform of various therapeutic agents, such as mRNA and drugs, for disease treatment 68-72. The SILY-EVs could be a new delivery platform with more efficiency for RNA and drug delivery in clinical applications.

Currently, the isolation of EVs is a very laborious process, especially the isolation of MSC-derived EVs 73, 74. Hence, the SILY-EVs designed in this study hold the promise for reducing the dosage of injected EVs in the therapeutic applications. In addition, large-scale production of SILY-EVs is significant for translational applications. We are currently actively evaluating several new approaches to achieve the large-scale production of SILY-EVs. Firstly, we are trying to improve the large-scale production of EVs via different 3D cell culture approaches, such as hollowfiber bioreactors and microcarriers in the spinner flask. These new methods once optimized may result in significantly higher yield of EVs, compared to the conventional 2D culture flasks used in the current study. Secondly, we are developing new EV characterization technologies, such as ExoView and Super-Resolution Microscope, to further optimize the conjugation process and improve the conjugation efficiency of SILY on EVs. Lastly, the SILY conjugation strategy designed in this study can be widely applicable to EVs derived from other sources. Hence, in the future this technology can be applied to EVs that can be produced in high yield and achieve large-scale of SILY-EVs.

## Conclusion

To address the current challenge of the locally injected EVs, in this study, we developed a new generation of collagen-binding EVs, SILY-EVs, that possessed stronger binding affinity to collagen and presented longer retention and higher therapeutic efficacy in the mouse hind limb ischemia model, compared to the free EVs. The SILY-EVs do not only hold promise to improve therapeutic efficacy of EV-based treatments in clinical applications, but also provides an innovative way to advance the EV applications in tissue engineering and regenerative medicine fields including functionalizing collagen-based biomaterial and delivering therapeutic agents.

## Methods

### Cell culture

MSCs were isolated from early gestation placental chorionic villus tissue (PMSCs) as we previously described 75, 76. PMSCs were expanded in D5 medium, including high-glucose DMEM (HyClone), 5% fetal bovine serum (FBS, HyClone), 20 ng/mL recombinant human basic fibroblast growth factor (bFGF, R&D systems), 20 ng/mL recombinant human epidermal growth factor (EGF, R&D systems), 100 UI/mL of penicillin and 100 μg/mL of streptomycin and incubated at 37^◦^C, 5% CO_2_. PMSCs were used between P3 and P5 for all experiments. ECFCs were isolated from human umbilical cord blood (HECFCs) as we previously described 77, 78. HECFCs were expanded in EGM-2 medium (Lonza). HECFCs were used between P4 and P6 for all experiments. Human THP-1 monocytes were obtained from our lab's cell bank and maintained in RPMI-1640 medium (HyClone) containing 10% FBS as described in our previous study 78.

### SILY-EV preparation

EVs were isolated from PMSCs as described in our previous study 24. Briefly, PMSCs were seeded at 2 × 10^4^ cells/cm^2^ in tissue culture-treated T175 flasks in 20 mL of EV-depleted FBS containing D5 medium for 48 h at 37^◦^C, 5% CO_2_. Conditioned medium was collected and sequentially centrifuged at 300 g for 10 min, 2,000 g for 20 min, and passed through a 0.22 mm filter. Then, the medium was concentrated using Amicon Ultra Centrifugal Filter Units with a 100-kDa MW cutoff (Sigma), transferred to thick wall polypropylene tubes (Beckman Coulter), and centrifuged at 8,836 g using the SW28 rotor and L7 Ultracentrifuge (Beckman Coulter). The supernatant was transferred to fresh tubes, centrifuged at 112,700 g for 90 min, and the pellet was resuspended in PBS (HyClone) and spun again at the same speed and time. The final pellet was resuspended in 10 mL of PBS per T175 flask and stored in aliquots at -80^◦^C.

For the SILY-EV preparation, SILY was conjugated to the EV surface by using click chemistry 26. Briefly, reactive dibenzylcyclootyne (DBCO) groups were incorporated in amine-containing proteins on EVs by using a heterobifunctional linker. Specifically, dibenzocyclooctynesulfo-N-hydroxysuccinimidyl ester (DBCO-sulfo-NHS) (Sigma) was added to EVs in PBS for reaction on a rotating mixer at room temperature for 2 h. Unconjugated DBCO-sulfo-NHS was removed by three washing steps using 100-kDa ultrafiltration tubes (Millipore). SILY peptide with an azide group on the lysine (SILY-azide) were synthesized by SciLight Biotechnology Co. The DBCO-conjugated EVs (DBCO-EVs) were then conjugated to the SILY-azide peptide via copper-free click chemistry. Specifically, SILY-azide peptide was added to DBCO-EVs in PBS, and the reaction was conducted on a rotating mixer at 4^◦^C for 12 h. The unincorporated peptides were removed by three washing steps on 100-kDa ultrafiltration tubes, and the SILY-EVs were resuspended in PBS. All the reactions of this conjugation approach were performed in neutral aqueous buffers without catalysts to void EV damage.

To assess the SILY conjugation qualitatively, TAMRA labeled SILY-azide (Lumiprobe Co.) was conjugated to the PKH67 (System Biosciences) labeled EVs with or without DBCO-sulfo-NHS linker. The conjugated EVs were applied to coverslips and imaged by fluorescence microscopy (Nikon) with a 60 objective.

ExoView tetraspanin kit assay was used to quantificationally measure the SILY conjugation efficiency on EVs. ExoView kits (NanoView Biosciences) were used according to the manufacturer's protocol. All chips were stored at 4^◦^C when not in use and allowed to warm to room temperature prior to use. Chips were pre-scanned using the provided protocol to identify any previously adhered particles during manufacturing. TAMRA-labeled SILY-EVs were diluted to a concentration 5 × 10^8^ particles/mL in the provided incubation solution, and 35 μL of sample were carefully pipetted onto the center of the chip. Samples were incubated on the chip overnight at room temperature. Following incubation, 1 mL of incubation solution was added and shaken at 500 rpm for 3 min at room temperature. 750 μL of incubation solution was removed and 750 μL of fresh solution was added, and the chip was once against shaken for 3 min. This step was repeated two more times to allow for thorough washing of the chip. After the last wash, 750 μL of incubation solution was removed and 250 μL of fluorescently labeled primary antibody CF647-anti-CD63 (clone: H5C6) was added (1:500 dilution in blocking solution). Chips were incubated with the antibodies for 1 h at room temperature while being shaken at 500 rpm. After incubation, chips were washed with 500 μL of incubation solution twice and with 750 μL rinse solution three times. Chips were washed once in DI water and carefully dried on an absorbent paper. Once fully dried, the chips were scanned using the ExoView® R100 (Nanoview Biosciences) for data acquisition.

### SILY-EV characterization

The concentration and size distribution of EVs and SILY-EVs were characterized by using NTA. EVs or SILY-EVs were diluted in triple-filtered (0.22 μm) MilliQ-water to reach the appropriate concentration range of 1 × 10^8^ - 1 × 10^9^ particles/mL. The diluted EVs or SILY-EVs were analyzed using the NanoSight LM10 (Malvern Panalytical) equipped with a 404-nm laser and sCMOS camera. Three 90-s videos were recorded for each sample at camera level 12 using NTA v.3.0 software. Data were consistently analyzed with a detection threshold of 3 and screen gain of 10.

The structure and morphology of EVs and SILY-EVs was characterized by cryo-EM. Briefly, Carbon EM grids (Ted Pella Inc.) were glow discharged at 30 mA, 30 s (Pelco Auto Sputter Coater SC-7, Ted Pella Inc.). 4 μL of 1 × 10^11^ EVs or SILY-EVs/mL solution was applied to the carbon side of the EM grid, blotted for 5 seconds, and then plunge-frozen into precooled vat of liquid ethane with Vitrobot Mark MkIII (FEI). Vitrified samples were then imaged with the Glacios Cryo Transmission Electron Microscope equipped with K3 Direct Electron Detector and acquired with SerialEM software (D.Mastronarde, Boulder Lab).

The protein contents of EVs and SILY-EVs were measured by using BCA assay. 5 × 10^10^ EVs or SILY-EVs were resuspended in RIPA buffer (Sigma-Aldrich) with protease inhibitors cocktail (Sigma-Aldrich), and then sonicated for 5 min. Protein contents were measured using a BCA protein assay kit (Thermo Scientific) according to the manufacturer's instruction.

The expression of EV typical markers on EVs and SILY-EVs were determined by using western-blot analysis. EV or SILY-EV pellets were lysed in RIPA lysis buffer containing protease inhibitor cocktail (Roche). 2 μg of protein per lane was loaded on a 4-12% graded Tris-glycine SDS-polyacrylamide gel (Invitrogen), then the proteins were transferred to a 0.22 μm PVDF membrane (Life Technologies). Membranes were blocked in 5% milk in PBS with 0.05% Tween-20 for 1 h at room temperature with shaking, and then incubated with primary antibodies anti-ALIX, anti-TSG101, anti-CD9 (Millipore), anti-CD63 (Thermo Fisher Scientific) and anti-calnexin (Cell Signaling Technology) at 1:1000 dilution in 5% milk in PBS at 4^◦^C overnight. Secondary antibody mouse IgGκ-HRP was diluted at 1:5000 in 5% milk in PBS and incubated at 1 h at room temperature with shaking (Santa Cruz Biotechnology). Proteins were visualized with Supersignal West Pico PLUS chemiluminescent substrate (ThermoFisher) using a KwikQuant imager (Kindle Biosciences).

### Cell uptake assay

EVs and SILY-EVs were labeled with DiD dye (ThermoFisher Scientific). DiD at a concentration of 2 μM was incubated with 1 × 10^10^ EVs or SILY-EVs for 1 h. Excess DiD was removed using 100-kDa ultrafiltration tube. 1 × 10^9^ DiD-EVs or DiD-SILY-EVs were added to each well and incubated with the HECFCs grown in µ-Slide 8 Well (ibidi) for 4 h or 16 h. In the control groups, a mixture of DiD-labelled (10%) and non-labelled (90%) EVs or SILY-EVs were incubated with the HECFCs for 4 h or 16 h. Media with DiD-EVs or DiD-SILY-EVs was aspirated, and the cells were fixed in 4% Paraformaldehyde (PFA, ThermoFisher Scientific) for 20 min. After washing with PBS, the cells were blocked in 1% Bovine Serum Albumin (BSA, Thermo Fisher Scientific) for 1 h at room temperature and incubated overnight with primary antibody anti-CD31 (Abcam) at 1:100 dilution in 1% BSA at 4^◦^C. After being washed with PBS 3 times, the tissue was incubated with the relevant secondary antibody (Life Technologies) at 1:500 dilution in PBS for 1 h at room temperature, and then nuclei were stained with 4',6-diamidino-2-phenylindole (DAPI, Thermo Fisher Scientific). After being washed with PBS 3 times, the images were captured using the Zeiss Observer Z1 microscope. Quantification of the number of cells with internalized DiD-EVs or DiD-SILY-EVs was performed using the ImageJ software (NIH). For the *in vitro* time-course experiments of EVs or SILY-EVs internalized in HECFCs, 1 × 10^9^ DiD-EVs or DiD-SILY-EVs were added to each well and incubated with the HECFCs grown in µ-Slide 8 Well for 16 h. Media with DiD-EVs or DiD-SILY-EVs was replaced with media without DiD-EVs and DiD-SILY-EVs. The HECFCs were cultured for another 48 h, then the cells were fixed in 4% PFA for 20 min and stained with DAPI. The images were captured using the Zeiss Observer Z1 microscope. Quantification of the number of cells with internalized DiD-EVs or DiD-SILY-EVs was performed using the ImageJ software.

### Tube formation assay

HECFCs were seeded onto growth factor reduced Matrigelcoated (Corning) in 96-well plates at 1 × 10^4^ cells/well and cultured in 100 μL of EBM-2 medium (Lonza) with EVs or SILY-EVs at 1 × 10^9^ particles/mL and incubated at 37^◦^C and 5% CO_2_ for 6 h. Images were taken using the Carl Zeiss Axio Observer D1 inverted microscope. The total segment length was quantified using ImageJ Angiogenesis Analyzer tool.

### HECFC proliferation assay

HECFCs were seeded in 96-well plates at 2 × 10^3^ cells/well and cultured in 100 μL of EGM-2 medium with EVs or SILY-EVs at 1 × 10^9^ particles/mL and incubated at 37°C and 5% CO_2_ for 5 d. Cell proliferation was assessed using CellTiter 96® AQueous One Solution Cell Proliferation Assay (MTS, Promega) according to the manufacturer's instructions.

### RT-qPCR

RNA extraction from cells or tissue was performed using RNeasy Mini Kit (Qiagen) according to the manufacturer's instructions, and cDNA was synthesized using Superscript II Reverse transcriptase (ThermoFisher Scientific). PCR was performed using the Biorad CFX96 Real-Time PCR Detection System (BioRad Laboratories) machinewith the SsoAdvanced SYBR Green Supermix (Bio-Rad). Amplification conditions after an initial denaturation step for 90 s at 95^◦^C were 40 cycles of 95^◦^C, 10 s, for denaturation, 55^◦^C, 10 s, for annealing and 72^◦^C, 30 s, for elongation. GAPDH was used as the reference gene for calculations. Data were analyzed by the delta-delta cycle threshold (Ct) method. Primer sequences used in this study are listed in **Table S1**.

### SILY-EV attachment on collagen surface

EVs were labeled with PKH67. Briefly, PKH67 was diluted 1:500 in PBS and added to EVs. The sample was mixed gently by flicking the tube and incubated at 37^◦^C for 10 min. Excess dye was removed by centrifuging at 16,000 g for 3 min at 4^◦^C. The EV pellets were resuspended in 500 μL of cold PBS, placed on ice for 5 min, and centrifuged again at 16,000 g for 3 min at 4^◦^C. Washes were repeated for a total of 3 times. After the final wash, labeled EVs were resuspended in 500 μL of PBS.

For the EV attachment assay, the wells in a 48-well cell culture plate were coated with 150 μL of 100 μg/mL type I collagen (Millipore) or type III collagen (Millipore) and incubated for 1 h at 37^◦^C. Then, the wells were washed three times with PBS, and 5 × 10^6^ PKH67-labeled EVs or SILY-EVs suspended in PBS was added into each well respectively and incubated for 1 h at 37^◦^C and 5% CO_2_. Then the wells were washed three times with PBS, and the attached EVs were imaged using a Carl Zeiss Axio Observer D1 inverted microscope. Quantification of images was performed using the ImageJ software. To further evaluate the EV attachment by using NTA, the wells in a 6-well cell culture plate were coated with 1.5 mL of 100 μg/mL type I collagen or type III collagen and incubated for 1 h at 37^◦^C. Then, the wells were washed three times with PBS, and 2 mL 5 × 10^7^/mL EVs or SILY-EVs suspended in PBS was added into each well respectively and incubated for 1 h at 37^◦^C and 5% CO_2_. The solution in each well was collected, and the remaining EVs or SILY-EVs were measured by using NTA.

### Macrophage modulation assay *in vitro*

EVs or SILY-EVs was added into the collagen coated wells of the 48-well cell culture plate at a density of 1 × 10^7^/cm^2^ and incubated for 10 min at 37^◦^C and 5% CO_2_, then the wells were washed 3 times with PBS. Human THP-1 monocytes were differentiated to macrophage-like THP-1 cells by stimulating with phorbol-12-myristate-13-acetate (PMA, Sigma) as described in the previous study 79. 2 × 10^4^ macrophage-like THP-1 cells/well activated with 100 ng/mL LPS (Sigma-Aldrich) were seeded in the collagen coated wells decorated with EVs or SILY-EVs respectively. After incubation for 12 h, cells were collected for evaluation of the gene expression related to inflammatory responses by using RT-qPCR assay as the procedure described above.

### Cell apoptosis assay *in vitro*

EVs or SILY-EVs was added into the collagen coated wells of the 96-well cell culture plate at a density of 1 × 10^7^/cm^2^ and incubated for 10 min at 37^◦^C and 5% CO_2_, then the wells were washed 3 times with PBS. For cell viability assay, 1 × 10^4^ HECFCs/well were seeded in the collagen coated wells decorated with EVs or SILY-EVs respectively. The ischemic simulated environment was set up as EGM-2 with 1% fetal bovine serum (FBS) and low concentration growth factors (1/10 of the original bulk in the EGM-2 bullet kit) at 1% O_2_, 37^◦^C and 5% CO_2_ as described previously 75. The HECFCs were cultured for 5 d and determined using LIVE/DEAD® Viability/Cytotoxicity Kit (Molecular Probes). Stained constructs were imaged using the Zeiss Observer Z1 microscope, and images further processed with ImageJ software. For cell survival assay, 1 × 10^4^ HECFCs/well were culture in the collagen coated wells decorated with EVs or SILY-EVs respectively in ischemic-mimicking hypoxic environment for 5 d and determined using MTS according to the manufacturer's instruction. For caspase 3 and caspase 9 assay, the cells were cultured in ischemic-mimicking hypoxic environment for 6 h, then lysed and analyzed by using a Caspase 3 Assay Kit and Caspase 9 Assay Kit (Cell Signaling Technology) according to the manufacturer's instruction. Fluorescence (ex 380 nm/em 450 nm) was measured using a SpectraMax i3x Multi-Mode Detection Platform (Molecular Devices).

### Angiogenic capacity assays *in vitro*

EVs or SILY-EVs was added into the collagen coated wells of the 96-well cell culture plate at a density of 1 × 10^7^/cm^2^ and incubated for 10 min at 37^◦^C and 5% CO_2_, then the wells were washed three times with PBS. For HECFC proliferation assay, 2 × 10^3^ HECFCs/well were seeded in the collagen coated wells decorated with EVs or SILY-EVs respectively and cultured for 5 d and determined using MTS assay according to the manufacturer's instruction. For Ki67 staining, 2 × 10^3^ HECFCs/well were seeded in the collagen coated wells decorated with EVs or SILY-EVs respectively and cultured for 5 d, and the cells were fixed in 4% PFA for 20 min. After being washed with PBS, the cells were blocked in 1% BSA and incubated overnight with primary antibody anti-ki67 (Abcam) at 1:100 dilution in 1% BSA at 4^◦^C. After being washed with PBS 3 times, the tissue was incubated with the relevant secondary antibody at 1:500 dilution in PBS for 1 h at room temperature, and then nuclei were stained with DAPI. After being washed with PBS 3 times, the images were captured using the Zeiss Observer Z1 microscope. The number of Ki67 positive cells was quantified by using ImageJ software. For evaluation of pro-angiogenic gene expression, 2 × 10^4^ HECFCs/well were seeded in the collagen coated 48-well plate decorated with EVs or SILY-EVs respectively. After incubation for 12 h, the HECFCs were collected for RT-qPCR assay as the procedure described above.

### *Ex vivo* retention study

Mouse ischemic hind limb tissue sections were blocked in 1% BSA for 1 h at room temperature and incubated overnight with primary antibody anti-collagen I (Abcam) at 1:100 dilution in 1% BSA at 4^◦^C. After being washed with PBS 3 times, the tissue was incubated with the relevant secondary antibody (Life Technologies) at 1:500 dilution in PBS for 1 h at room temperature, and then nuclei were stained with DAPI. 1 × 10^7^ EVs or SILY-EVs labeled with DiD were incubated with the mouse ischemic hind limb tissue sections stained with CD31 and DAPI for 1 h at room temperature, followed by washing with PBS 3 times. The images were captured using the Zeiss Observer Z1 microscope. Six views were captured from each slice, and six slices from different mice were used in this study. Quantification of the number of EVs or SILY-EVs attached on the collagen was performed using the ImageJ software.

To further evaluate the retention of EVs or SILY-EVs on the ischemic hind limb tissue sections at different time points, 1 × 10^7^ EVs or SILY-EVs labeled with DiD were incubated with the mouse ischemic hind limb tissue sections stained with CD31 and DAPI for 1 h at room temperature, followed by incubating with PBS at 37^◦^C. The images were captured at day 1, 3 and 7 using the Zeiss Observer Z1 microscope. Quantification of the number of EVs or SILY-EVs attached on the collagen was performed using the ImageJ software.

### Mouse hind limb ischemia model

All animal experiments were approved by the Institutional Animal Care and Use Committee at University of California Davis. Female mice (C57BL/6J, 8-wk-old) were obtained from the Jackson Laboratory. Under anesthesia, mice were subjected to unilateral hind limb ischemia surgeries as previously described 80. The mice were shaved and prepped, then the left femoral artery and vein were exposed and dissected from the femoral nerve, and the proximal portion of the femoral artery and vein were ligated with 6-0 silk sutures. The distal portion of the saphenous artery and vein and the remaining collateral arterial and venous side branches were ligated and completely excised from the hind limb. The overlying skin was closed using Nexaband veterinary glue (Abbott Animal Health).

### *In vivo* retention study

To evaluate the retention of EVs and SILY-EVs in the mouse hind limb ischemia model, the mice were randomly assigned to two groups: (1) EVs (n = 6) and (2) SILY-EVs (n = 6). EVs or SILY-EVs were labeled with DiD, and 100 μL 1 × 10^11^/mL DiD-labeled EVs or SILY-EVs was intramuscularly injected into four different sites around the hind limb ischemic region in the previous studies 81, 82. Mice under anesthesia were imaged at multiple time points up to day 7 using the SPECTRAL Lago X Imaging System (Xenogen, Caliper Life Sciences). The injured area was selected as the region of interest (ROI), and the radiant efficiency was calculated within the ROIs. The outcomes were blindly assessed.

### *In vivo* treatment study

To evaluate the effects of EVs and SILY-EVs on inflammatory response, revascularization and muscle repair in the mouse hind limb ischemia model, the mice were randomly assigned to three groups: (1) PBS (n = 6); (2) EVs (n = 6); (3) SILY-EVs (n = 6). 100 μL 1 × 10^11^/mL EVs or SILY-EVs was intramuscularly injected into four different sites around the hind limb ischemic region as described above.

### Blood perfusion assessment

For the blood perfusion assessment, the ischemic limb-to-nonischemic limb blood perfusion ratio was measured weekly up to 3 weeks using a Laser Doppler perfusion imager (Moor Instruments), which provides noninvasive measurement of the blood flow by determining the Doppler frequency shift for light reflected off the moving red blood cells. Briefly, the mice under anesthesia were secured on a monochromatic surface, and an area was scanned from the lower abdomen to the end of the toes. Color images were obtained, and the hindlimb perfusion ratios were determined by comparing the perfusion of the injured hind limbs and the contralateral normal hind limbs. The outcomes were blindly assessed.

### Micro-CT imaging

The mice were euthanized at day 21, and a catheter was inserted into the left ventricle of the mice. The mouse vasculature was flushed with PBS supplemented with 100 U/mL heparin sodium (Fresenius Kabi) and then 4% PFA. One to two milliliters of Microfil MV-120 (Flow Tech, Inc.) was injected into the left ventricle to increase contrast of vasculature. The leg muscles from both limbs were separately collected and imaged with a micro-CT imaging system (VivaCT40; Scanco Medical) using a voxel size of 10.5 mm, X-ray source voltage of 55 kVp, and 145 mA current. The micro-CT images were analyzed using a matching three-dimensional segmentation algorithm of thresholding.

### Histology

All mice were euthanized at day 21, and the hind limbs were harvested and trimmed to dissociate out the upper part of ligation. Tissues were immersed in 4% PFA for 48 h at 4^◦^C. Cryosections (thickness = 6 μm) were prepared. For histological analysis, the sections were stained by H&E and Masson's trichrome (Thermo Fisher Scientific) according to the manufacturer's instructions respectively. For immunohistochemistry staining, the tissue sections were incubated with different primary antibodies anti-CCR7 (Abcam), anti-CD163 (Abcam), anti-CD68 (Abcam), anti-CD31 (Abcam), or anti-α-SMA (Abcam) at 1:100 dilution in 1% BSA overnight at 4°C. After being washed with PBS 3 times, the tissue sections were incubated with the relevant secondary antibody at 1:500 dilution in PBS for 1 h at room temperature, and then nuclei were stained with DAPI. After being washed with PBS 3 times, the images were captured using the Zeiss Observer Z1 microscope. The images were analyzed by using ImageJ software.

### Statistical analysis

All the outcomes in this study were assessed blindly. Graphs and statistical analysis were performed using Prism. For multiple-sample comparison, analysis of variance (ANOVA) was used to evaluate whether a significant difference existed between groups with different treatments, and a multiple comparison procedure Holm's *t* test was used for post- analysis. The values of P < 0.05 indicates significant difference between samples in comparison.

## Supplementary Material

Supplementary figures and table.
