# Supplementary material for: Engineered extracellular vesicles with high collagen-binding affinity present superior in situ retention and therapeutic efficacy in tissue repair
Source: Theranostics. 2022 Aug 8;12(13):6021–37. doi: 10.7150/thno.70448 (PMC9373818; doi:10.7150/thno.70448)
Supplement: Supplementary file 1 — Supplementary figures and table. [file thnov12p6021s1.pdf]

Supplementary materials

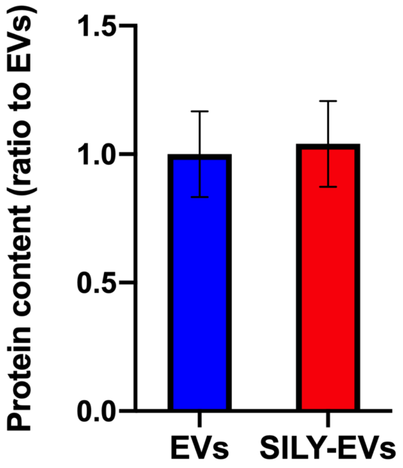

Figure S1. BCA assay of the same number of EVs and SILY-EVs. Data are expressed as mean ± standard deviation (n = 6).

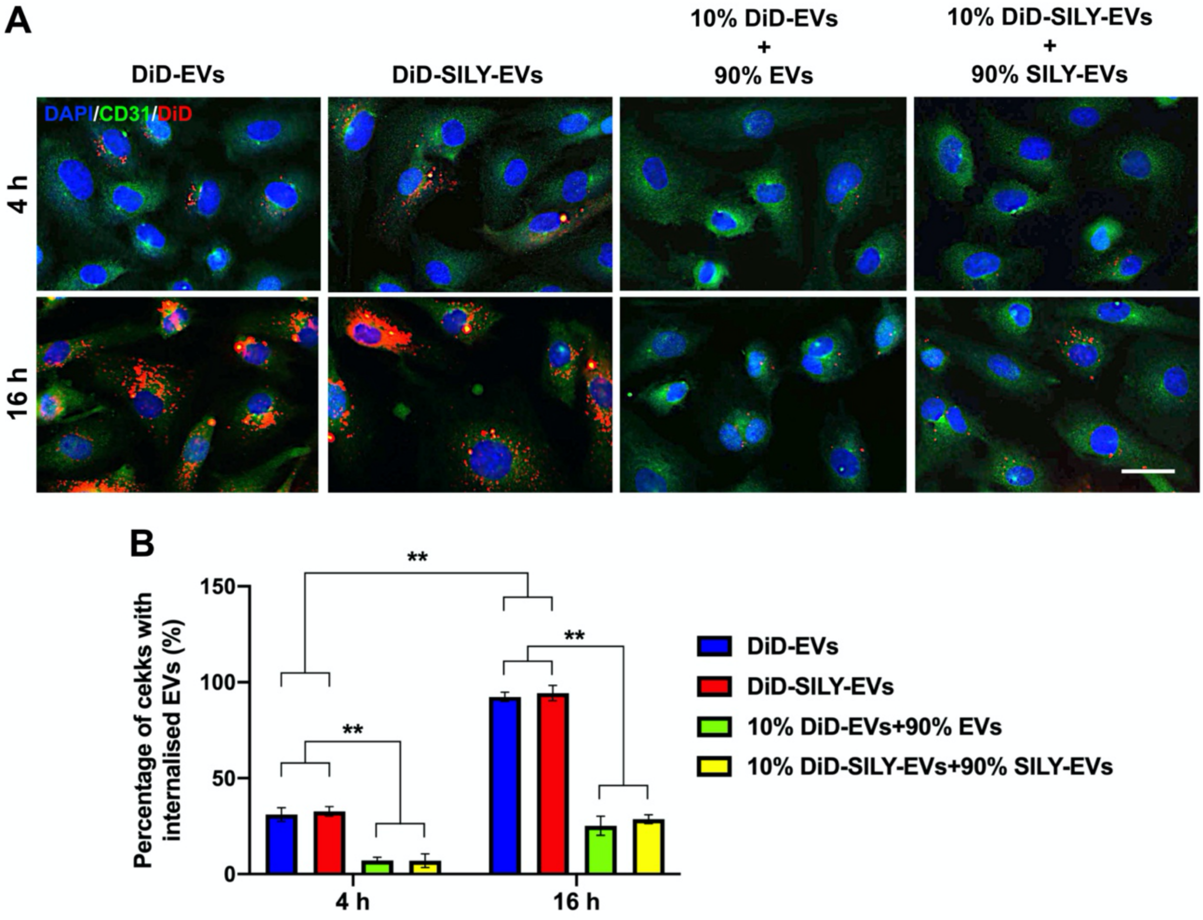

Figure S2. Cellular uptake efficiency of EVs and SILY-EVs. (A) Images of uptake of DiD-labeled EVs or SILY-EVs in HECFCs. Scale bar = 10 μm. (B) Quantification of the uptake rate of EVs or SILY-EVs in HECFCs. Data are expressed as mean ± standard deviation: $^{**}p < 0.01$ ($n = 6$).

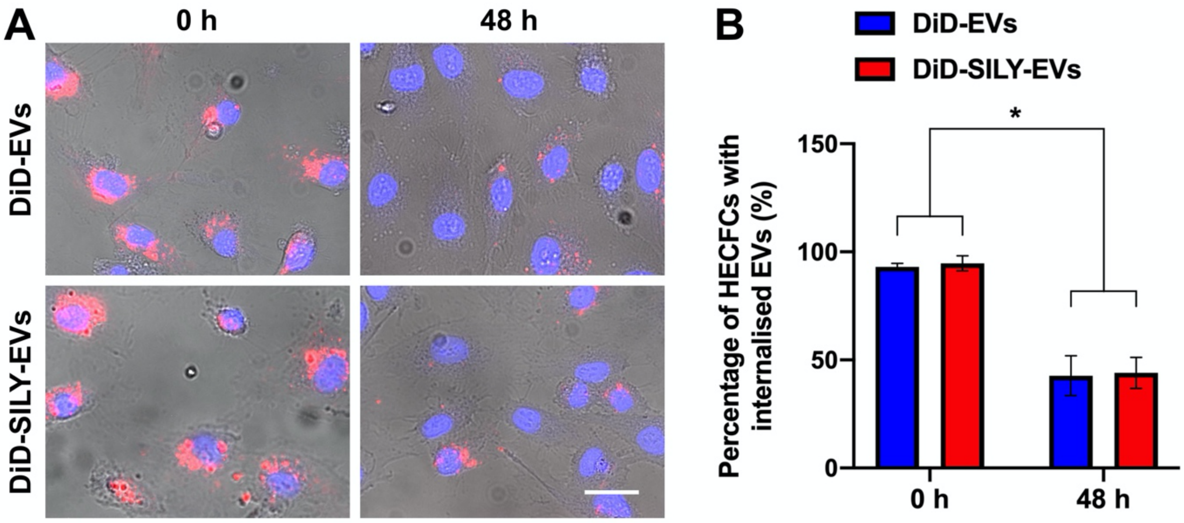

Figure S3. Kinetics of EVs and SILY-EVs internalized in HECFCs. (A) Images of DiD-labeled EVs or SILY-EVs internalized in HECFCs cultured in medium without DiD-labeled EVs or SILY-EVs for 0 h and 48 h. Scale bar = 10 μm. (B) Quantification of the percentage of HECFCs with internalized DiD-labeled EVs or SILY-EVs. Data are expressed as mean ± standard deviation: $*p < 0.05$ ($n = 6$).

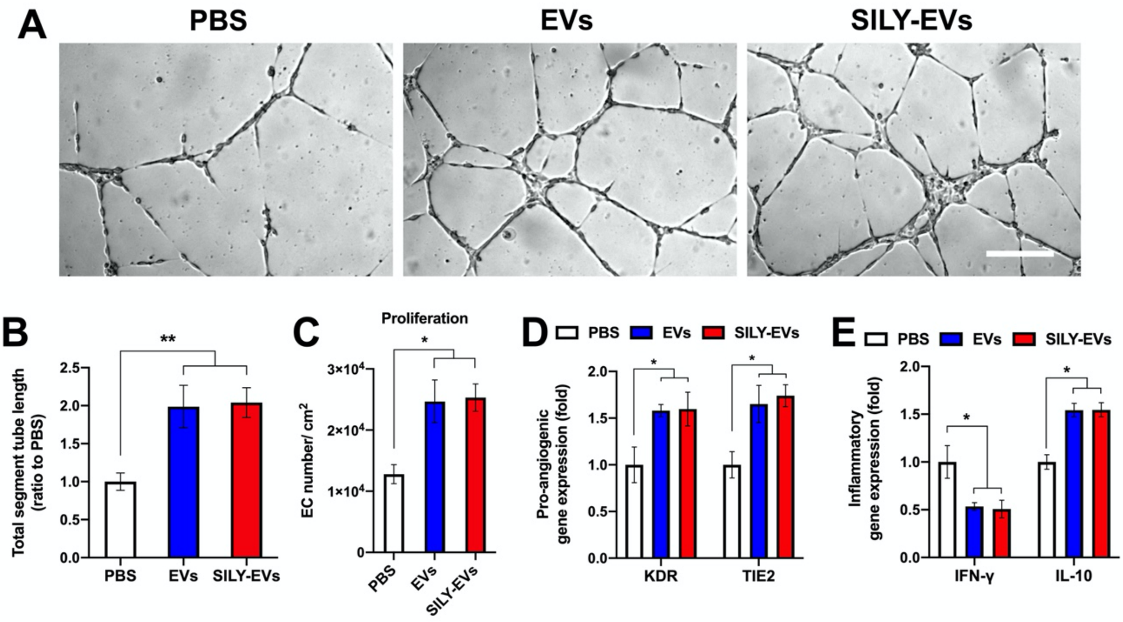

Figure S4. Biological functions of EVs and SILY-EVs. (A) Effect of EVs or SILY-EVs on tube formation. Scale bar = 10 μm. (B) Quantification of total segment tube length. (C) Effect of EVs or SILY-EVs on HECFC proliferation. (D) Effect of EVs or SILY-EVs on pro-angiogenic gene expression. (E) Effect of EVs or SILY-EVs on inflammatory gene expression. Data are expressed as mean ± standard deviation: *$p < 0.05$, **$p < 0.01$ ($n = 6$).

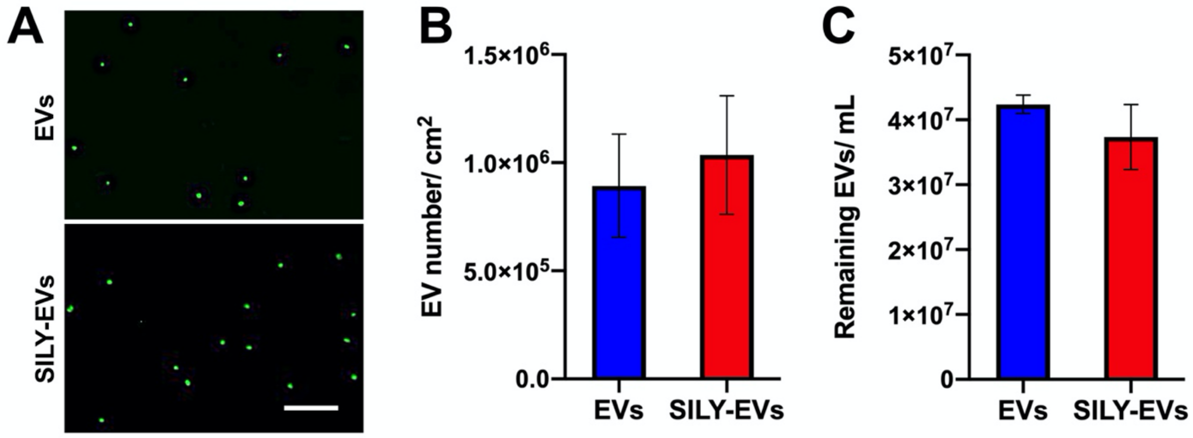

Figure S5. Attachment of EVs and SILY-EVs on type III collagen surface. (A) Images of attached PKH67-labeled EVs or SILY-EVs on type III collagen surface. Scale bar = 3 μm. (B) Quantification of the numbers of EVs or SILY-EVs attached to the type III collagen surface. (C) Quantification of the numbers of the EVs or SILY-EVs unattached to the type III collagen surface by using NTA. Data are expressed as mean ± standard deviation (n = 6).

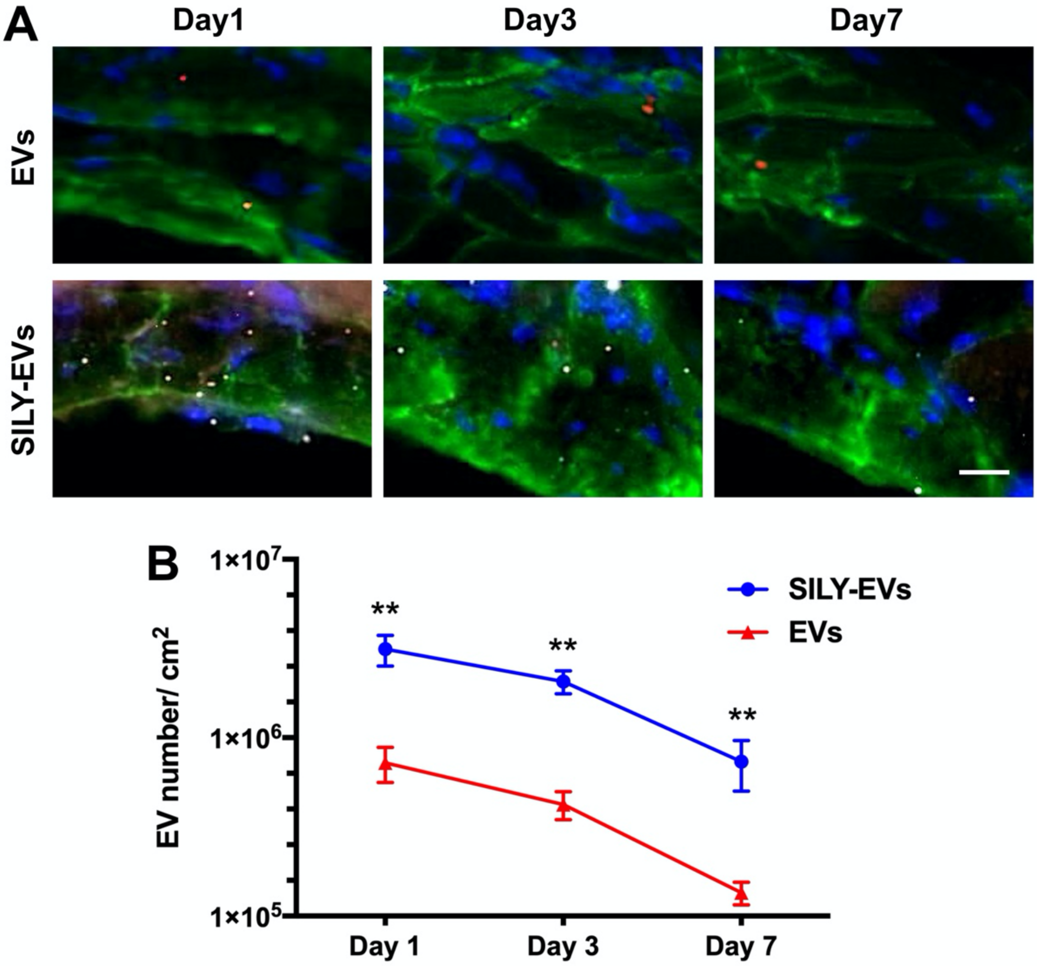

Figure S6. *Ex vivo* evaluation for retention of EVs and SILY-EVs on the ischemic hind limb tissue section. (A) Retention of EVs and SILY-EVs on the ischemic hind limb tissue section via binding to collagen at day 1, 3 and 7. Green indicated collagen, blue indicated nucleus, red indicated DiD-labeled EVs, and gray indicated TAMRA-labeled SILY. The white arrows indicated EVs or SILY-EVs attached on the collagen of the ischemic hind limb tissue section. Scale bar = 10 μm. (B) Quantification of the EVs and SILY-EVs attached on the collagen of the ischemic hind limb tissue section. Data are expressed as mean ± standard deviation: $^{**}p < 0.01$ ($n = 6$).

Gene	Forward sequence	Reverse sequence
hKDR	GGAACCTCACTATCCGCAGAGT	CCAAGTTCGTCTTTTCCTGGGC
hTIE2	GGTCAAGCAACCCAGCCTTTTC	CAGGTCATTCCAGCAGAGCCAA
hIFN-γ	GAGTGTGGAGACCATCAAGGAAG	TGCTTTGCGTTGGACATTCAAGTC
hIL10	TCTCCGAGATGCCTTCAGCAGA	TCAGACAAGGCTTGGCAACCCA
hGAPDH	GTCTCCTCTGACTTCAACAGCG	ACCACCCTGTTGCTGTAGCCAA
mIFN-γ	CAGCAACAGCAAGGCGAAAAAGG	TTTCCGCTTCCTGAGGCTGGAT
mIL10	CGGGAAGACAATAACTGCACCC	CGGTTAGCAGTATGTTGTCCAGC
mMyoz1	GGAACTTGGCATTGACCTACTG	AAACTTGGGCATCTGGAAGG
mMyoz3	TGGCAGCAGAAGTCACACTC	AGTTCCAAGCCACTGAAGGAC
mAngII	AACTCGCTCCTTCAGAAGCAGC	TTCCGCACAGTCTCTGAAGGTG
mPECAM1	CCAAAGCCAGTAGCATCATGGTC	GGATGGTGAAGTTGGCTACAGG
mGAPDH	CATCACTGCCACCCAGAAGACTG	ATGCCAGTGAGCTTCCCGTTCAG

Table S1. Primers used for RT-qPCR.
